# Silencing of *cyp-33C9* Gene Affects the Reproduction and Pathogenicity of the Pine Wood Nematode, *Bursaphelenchus xylophilus*

**DOI:** 10.3390/ijms20184520

**Published:** 2019-09-12

**Authors:** Xiuwen Qiu, Lili Yang, Jianren Ye, Wei Wang, Tiantian Zhao, Hao Hu, Guixiang Zhou

**Affiliations:** 1 Poyang Lake Eco-economy Research Center, Jiujiang University, Jiujiang 332005, Jiangxi, China; 2 College of Bioscience and Bioengineering, Jiangxi Agricultural University, Nanchang 330045, Jiangxi, China; 3 Office of Mountain-River-Lake Development Committee of Jiangxi Province, Nanchang 330046, Jiangxi, China; 4 College of Environment, Zhejiang University of Technology, Hangzhou 310014, Zhejiang, China; 5 College of Forestry, Nanjing Forestry University, Nanjing 210037, Jiangsu, China

**Keywords:** cytochrome P450s, gene silencing, RNA interference, pine wilt disease, *Bursaphelenchus xylophilus*

## Abstract

Cytochrome P450 genes are very important for plant-parasitic nematodes to reproduce and to metabolize xenobiotic compounds generated by their host plants. The pine wood nematode (PWN), *Bursaphelenchus xylophilus*, causes very high annual economic losses by killing large numbers of pine trees across Asia and into Europe. In this study, we used RNA interference (RNAi) to analyze the function of the *cyp-33C9* gene of PWN. Our results showed that expression of the *cyp-33C9* gene was suppressed successfully after soaking nematodes for 24 h in *cyp-33C9* double-stranded RNA (dsRNA). The silencing of the *cyp-33C9* gene significantly decreased the feeding, reproduction, oviposition and egg hatch of *B. xylophilus*. Meanwhile, the migration speed of *B. xylophilus* in *Pinus thunbergii* was reduced in the early stages when the *cyp-33C9* gene was silenced in the nematodes. Moreover, knockdown of the *cyp-33C9* gene in *B. xylophilus* caused a decrease in pathogenicity to pine trees. These results suggest that the *cyp-33C9* gene plays an important role in the reproduction and pathogenicity of *B. xylophilus*. This discovery identified several functions of the *cyp-33C9* gene in *B. xylophilus* and provided useful information for understanding the molecular mechanism behind pine wilt disease caused by PWN.

## 1. Introduction

Pine wilt disease (PWD) is one of the most serious diseases of forest trees, affecting conifers around the world [1]. As the causal agent of PWD, the pine wood nematode (PWN), *Bursaphelenchus xylophilus* (Steinr and Buhrer) Nickle, is a migratory plant endoparasite [2]. *B. xylophilus* is considered to be native to North America, where it causes little damage to the native pine species which are resistant to the nematode [3]. However, once introduced into a new area where no natural resistance is present in the conifers, *B. xylophilus* has caused the death of millions of pine trees, resulting in huge economic losses [4]. At the start of the 20th century, the PWN was introduced into Japan, from where it has subsequently spread to other countries in Asia, such as China and Korea [5,6,7]. *B. xylophilus* was first detected in Europe in 1999 and has now been found in Portugal and Spain [8,9].

As important components of a monooxygenase system, cytochrome P450s are widely distributed in virtually all living organisms, such as mammals, plants, microorganisms and invertebrates [10,11]. Cytochrome P450s catalyze not only the oxidative metabolism of xenobiotic compounds, such as pesticides, drugs, mutagens and plant toxins [12], but also the synthesis and degradation of endobiotic compounds, such as steroid hormones, amino acids and fatty acids [13,14,15]. In addition, cytochrome P450s have been associated with other functions, including cell growth, infection, reproduction and other processes [16]. For example, cytochrome P450s are vital for the correct execution of meiosis, eggshell development and anterior–posterior polarization of the embryo in *Caenorhabditis elegans* [17]. Ziniel et al. demonstrated that CYP3050A1 was essential for parasite survival and egg development in the blood fluke *Schistosoma mansoni* [18]. 

The genome of *B. xylophilus* was sequenced in 2011 and 76 cytochrome P450 genes were detected [19]. Pine trees produce a complex mixture of nematicidal and nematistatic compounds (such as terpenoids and cyclic aromatic compounds) in response to invasion by PWNs [20,21]. The cytochrome P450s of *B. xylophilus* are probably involved in the catabolism of these pine tree defense chemicals [19]. The detoxification of xenobiotics by the nematode can be divided into three phases: (I) the addition of functional groups to molecules, making them more suitable substrates for the next stage; (II) the execution of detoxification reactions; (III) the release of the metabolites [22]. Cytochrome P450s play the most important role in phase I [23]. With the development of molecular research technologies in recent decades, more and more functions of cytochrome P450 genes have been revealed [24]. However, the roles of cytochrome P450 genes in the pathogenic process of *B. xylophilus* are currently unknown. 

RNA interference (RNAi) is a powerful tool for investigating the biological functions of genes, as it causes the degradation of specific mRNA sequence by using homologous double-stranded RNA (dsRNA) to silence gene function at the post-transcriptional level [25,26,27]. Since it was first described in the free-living nematode *C. elegans* in 1998, the RNAi technique has been widely used to study gene functions in plant-parasitic nematodes, such as cyst nematodes (e.g., *Globodera* spp.), root-knot nematodes (e.g., *Meloidogyne* spp.) and migratory burrowing nematodes (e.g., *Radopholus similis*) [28,29,30]. In *B. xylophilus*, several essential genes, such as cellulase, arginine kinase, pectinase and heat shock protein 70 genes, have been silenced using dsRNA [31,32,33,34]. As a member of the cytochrome P450 gene family, the *cyp-33C9* gene has been reported in *Caenorhabditis briggsae, Caenorhabditis remanei, Caenorhabditis brenneri* and *B. xylophilus* according to the NCBI database (https://blast.ncbi.nlm.nih.gov/Blast.cgi). Up to now, the roles of the *cyp-33C9* gene in these nematodes have not been well characterized. 

Our previous study demonstrated that the expression of the *cyp-33C9* gene in the PWN was up-regulated 6.2-fold after *B. xylophilus* invaded pine trees, which indicated that the *cyp-33C9* gene plays an important role in the pathogenicity of the PWN [24]. Therefore, the aim of the present study was to examine the effects of silencing the *cyp-33C9* gene on the morphology, development, reproduction and pathogenicity of *B. xylophilus*. The results of this study will not only reveal the functions of *cyp-33C9* gene in *B. xylophilus*, but also provide useful information for elucidating the molecular mechanism of pathogenicity in the PWN. 

## 2. Results

### 2.1. Effects of RNA Interference (RNAi) on Feeding and Reproduction of Bursaphelenchus xylophilus

**Figure 1:**
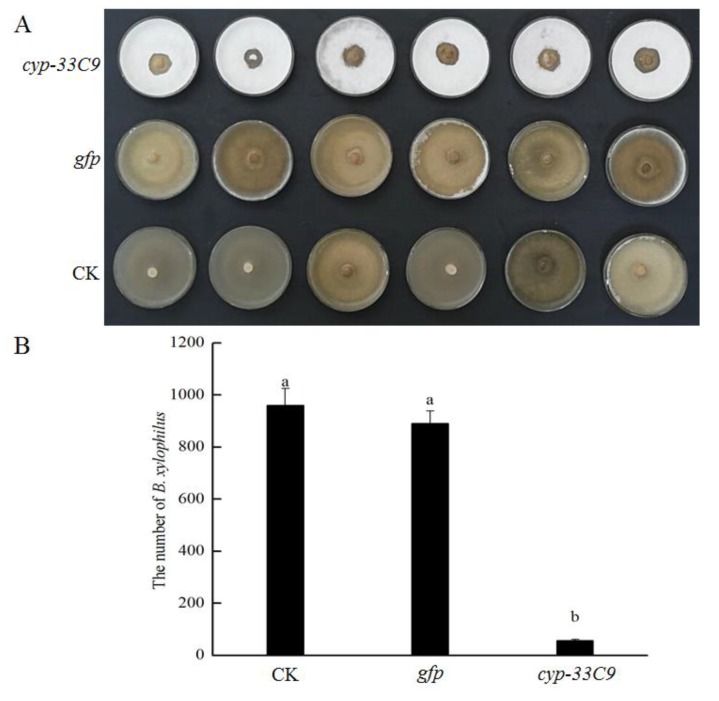
Effects of RNA interference (RNAi) on feeding and reproduction of *Bursaphelenchus xylophilus*. *Botrytis cinerea* cultures on potato dextrose agar (PDA) plates 5 d after inoculation with 200 s-stage juveniles (J2s) soaked in ddH_2_O (CK) or dsRNA (*gfp* or *cyp-33C9*) (**A**); the numbers of *B. xylophilus* washed from PDA plates of *B. cinerea* inoculated with *B. xylophilus* J2s treated with ddH_2_O (CK) or dsRNA (*gfp* or *cyp-33C9*). The error bars indicate standard deviations, and treatments with different letters indicate significant differences (*p* < 0.05) (**B**).

The experiments to study the effects of RNAi on feeding and reproduction of *B. xylophilus* were performed on *Botrytis cinerea* cultures on potato dextrose agar (PDA) plates. The green fluorescent protein (*gfp*) gene dsRNA treatment was used to evaluate the influence of non-endogenous dsRNA on feeding and reproduction of *B. xylophilus*. The nematodes soaked in *cyp-33C9* dsRNA solution showed significantly reduced population size compared with that of nematodes soaked in ddH_2_O (control, CK) or *gfp* dsRNA solution (Figure 1A). After five days incubation post-treatment on *B. cinerea* plates at 25 °C, the number of *B. xylophilus* second-stage juveniles (J2s) per plate after inoculation with J2s treated with *cyp-33C9* dsRNA, ddH_2_O (CK) or *gfp* dsRNA solution were 97,970 and 890, respectively (Figure 1B). These results suggested that feeding and reproduction of *B. xylophilus* were significantly suppressed by *cyp-33C9* dsRNA interference. 

### 2.2. Effects of RNA Interference (RNAi) on Oviposition of Bursaphelenchus xylophilus

**Figure 2:**
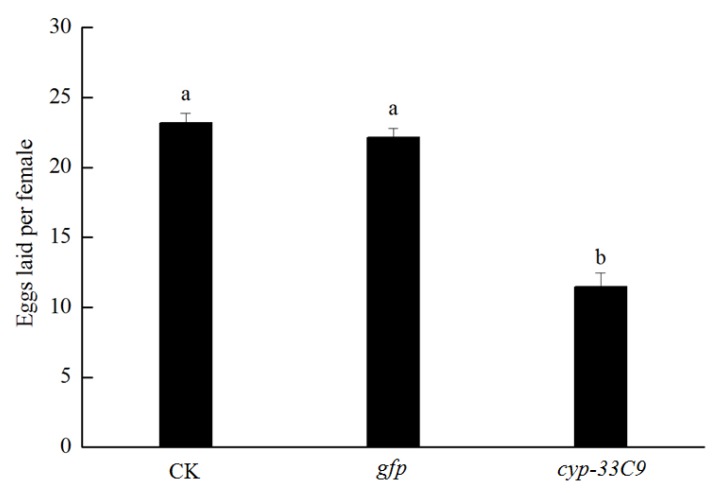
The oviposition of *Bursaphelenchus xylophilus* after RNA interference of *cyp-33C9*. The bars indicate standard deviation, and different letters indicate significant differences (*p* < 0.05) among treatments.

To further assess the influence of the *cyp-33C9* gene on the fecundity of *B. xylophilus*, the number of eggs laid per female nematode was determined. The results showed that the number of eggs laid per female nematode soaked in ddH_2_O and *gfp* dsRNA solution were 23 and 22, respectively (Figure 2). However, the number of eggs laid per female nematode soaked in *cyp-33C9* dsRNA solution was only 10, which was much fewer than that from nematodes treated with ddH_2_O or *gfp* dsRNA solution. These results indicated that the gene silencing of *cyp-33C9* by dsRNA interference has a significant inhibitory effect on oviposition of *B. xylophilus*. 

### 2.3. Effects of RNAi on Hatch of Bursaphelenchus xylophilus 

**Figure 3:**
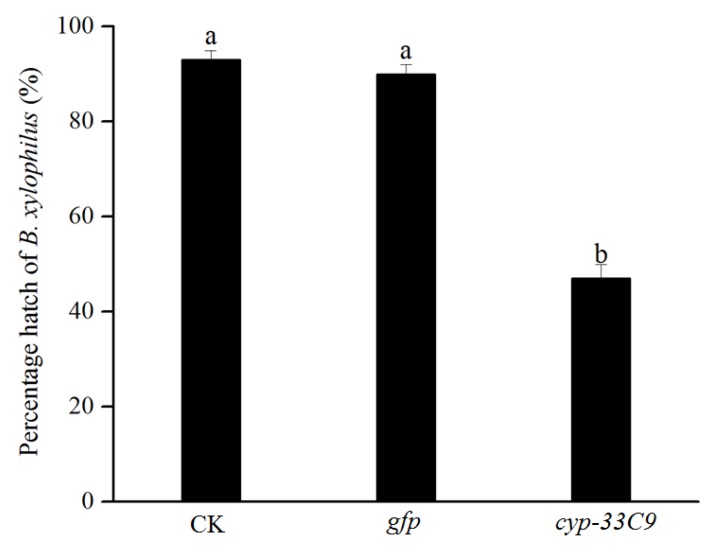
The percentage hatch of *Bursaphelenchus xylophilus* after RNA interference of *cyp-33C9*. The bars indicate the standard deviation, and different letters indicate significant differences (*p* < 0.05), according to Tukey’s test, among the treatments.

The effects of RNAi silencing of *cyp-33C9* on percentage hatch of *B. xylophilus* were similar to those on oviposition. The percentage hatch of *B. xylophilus* with *cyp-33C9* dsRNA treatment was 52%, which was lower than that with ddH_2_O or *gfp* dsRNA treatments (Figure 3). No significant differences were observed between ddH_2_O and *gfp* dsRNA treatments, indicating that the non-endogenous dsRNA had no obvious negative effect toward the nematodes.

### 2.4. Effects of RNA Interference (RNAi) on Individual Body Length of Bursaphelenchus xylophilus

**Figure 4:**
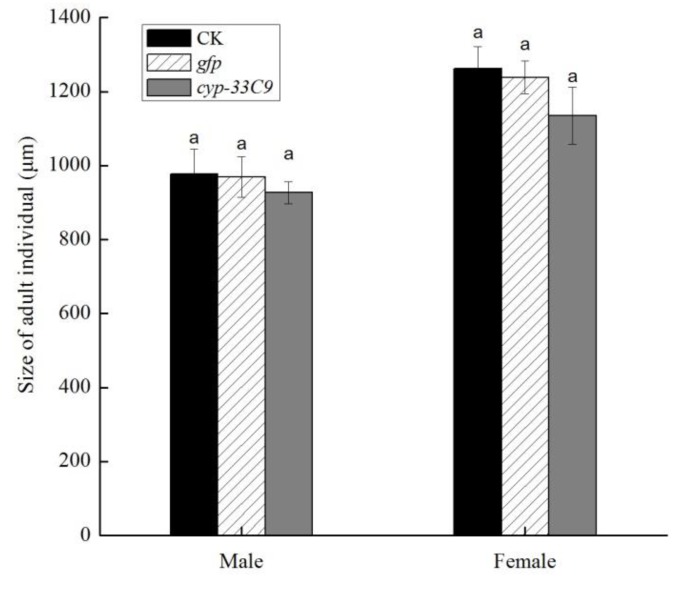
Effects of RNA interference on individual body length of *Bursaphelenchus xylophilus*. The bars indicate standard deviation, and different letters within a gender indicate significant differences (*p* < 0.05), according to Tukey’s test, among the treatments.

After being soaked in *cyp-33C9* dsRNA solution, the individual body length of both the male and female J2s was slightly but not significantly shorter (*p* > 0.05) than that following soaking in ddH_2_O or *gfp* dsRNA solution (Figure 4). The body length of *B. xylophilus* following ddH_2_O treatment was similar to that following *gfp* dsRNA treatment. No significant differences in body lengths between the three different treatments were observed, suggesting that *cyp-33C9* dsRNA interference had no significant effect on the individual body length of *B. xylophilus*. 

### 2.5. Determination of RNA Interference (RNAi) on Expression of cyp-33C9 in Bursaphelenchus xylophilus

**Figure 5:**
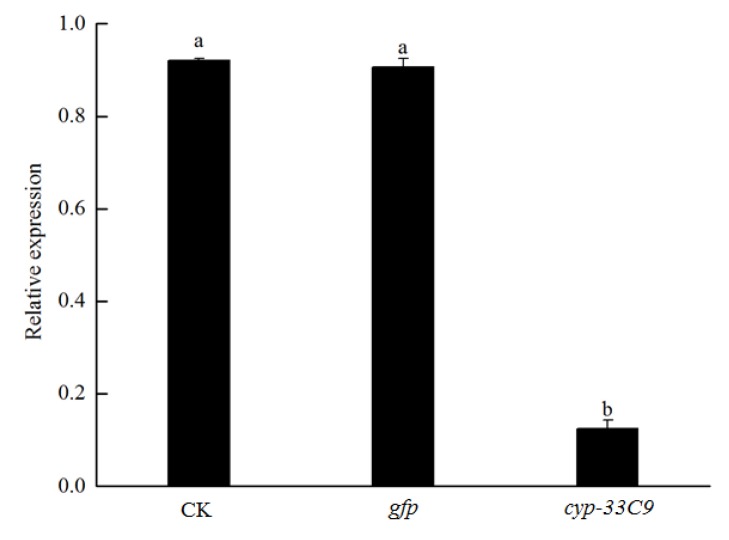
Quantitative reverse transcription PCR analysis of the RNA interference efficiency in *Bursaphelenchus xylophilus* after treatment with ddH_2_O or dsRNA (*gfp* or *cyp-33C9*). The bars indicate standard deviation, and different letters indicate significant differences (*p* < 0.05), according to Tukey’s test, among treatments.

Quantitative reverse transcription PCR (qRT-PCR) was performed to test the effects of RNAi on the expression levels of the *cyp-33C9* gene in nematodes soaked in target gene dsRNA solution, compared with the corresponding controls. The average expression level of the *cyp-33C9* gene in *B. xylophilus* with ddH_2_O and *gfp* dsRNA treatments were 0.92 and 0.91, respectively (Figure 5). However, the mean expression level of the *cyp-33C9* gene in nematodes soaked in target dsRNA solution was only 0.12 (*p* < 0.05). These results suggested that the *cyp-33C9* gene was silenced by dsRNA interference and that non-endogenous dsRNA had no influence on the expression of the target gene. 

### 2.6. Effects of RNA Interference (RNAi) on Pine Wood Nematode Migration and Reproduction of Bursaphelenchus xylophilus in Pinus thunbergii

In order to study the migration of pine wood nematodes in the host after silencing of the *cyp-33C9* gene by RNAi, we counted the number of *B. xylophilus* in different parts of pine trees at different sampling times following inoculation of J2s which had been treated with ddH_2_O or dsRNA. For ddH_2_O-treated J2s, there were 66 nematodes at 5 cm above the site of inoculation, but no nematodes existed at 10 or 15 cm above the site at day 5 after inoculation, whereas the number of nematodes at 5, 10 and 15 cm below the site of inoculation were 256, 57 and 9, respectively (Table 1). For *cyp-33C9* dsRNA treatment, the distribution of nematodes in *P. thunbergii* was significantly different from that in the ddH_2_O treatment. 

These results showed that, after being inoculated into *P. thunbergii*, *B. xylophilus* nematodes migrated in two directions (up and down), with the speed of upward migration being slower than that of downward migration. In addition, the migration speed of *B. xylophilus* treated with ddH_2_O in *P. thunbergii* was faster than those treated with *cyp-33C9* dsRNA (Table 1). At day 10 after inoculation, the nematodes treated with ddH_2_O or *cyp-33C9* dsRNA solution had migrated to all monitored sites of the pine trees, but the number of migrated nematodes in *P. thunbergii* following treatment with the *cyp-33C9* dsRNA was significantly lower than that of nematodes treated with ddH_2_O. This result showed that the migration speed of *B. xylophilus* treated with ddH_2_O in pine trees was faster than that of J2s treated with *cyp-33C9* dsRNA, indicating that *cyp-33C9* dsRNA treatment has a negative effect on the migration of *B. xylophilus*. The number of PWN J2s in *P. thunbergii* increased dramatically after 10 d of inoculation for all three treatments and reached the maximum at day 20 (Table 1). Interestingly, no significant difference was observed between the numbers of J2s from the three treatments at the different monitoring sites at 25 d after inoculation, presumably as the numbers of *cyp-33C9* dsRNA-treated J2s had caught up with the control J2s (Table 1). The migration speed of the control J2s was not significantly different from that of the *gfp* dsRNA-treated J2s throughout the study, indicating that non-endogenous dsRNA had no influence on the migration speed.

### 2.7. Effects of RNA interference (RNAi) on Pathogenicity of Bursaphelenchus xylophilus

**Figure 6:**
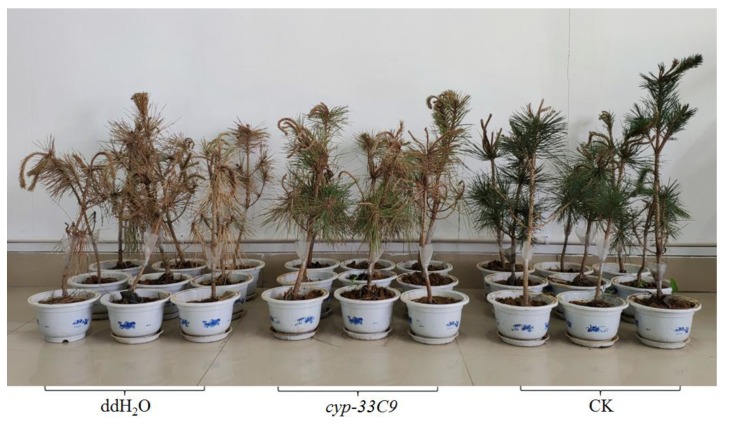
Wilting symptoms of *P. thunbergii* seedlings at 25 d after inoculation with *Bursaphelenchus xylophilus* soaked in ddH_2_O (“ddH_2_O”) or *cyp-33C9* dsRNA solution (“*cyp-33C9”*), respectively. Controls (“CK”) were represented by *P. thunbergii* seedlings inoculated with ddH_2_O alone (no nematodes).

After inoculated with *B. xylophilus* for 25 d, the symptoms in *P. thunbergii* were observed to determine whether treatment with RNAi affected the pathogenicity of the nematodes. Of the three nematode treatments, the 2-year-old *P. thunbergii* seedlings inoculated with *B. xylophilus* soaked in ddH_2_O appeared to wilt first, with the percentage needle wilting reaching 100% at 25 d after inoculation (Figure 6). At the same time, the percentage needle wilting of the *P. thunbergii* seedlings inoculated with *B. xylophilus* soaked in *cyp-33C9* dsRNA solution was significantly lower at 44%. In contrast, the CK *P. thunbergii* seedlings mock-inoculated with water (no J2s) grew well throughout the whole experiment process. These results indicated that silencing of the *cyp-33C9* gene decreased the percentage wilting of *P. thunbergii* seedlings and reduced the pathogenicity of *B. xylophilus*.

## 3. Discussion

The cytochrome P450s are considered to be involved in reproductive development in nematodes [17,18]. In the present study, we found that the *cyp-33C9* gene was silenced when *B. xylophilus* was soaked 1 μg·μL^−1^ in *cyp-33C9* dsRNA solution. Xu et al. observed a similar result (with gene expression of 0.05) when the *cyp-33C9* dsRNA solution was 800 ng·μL^−1^ [16]. These results indicated that different *cyp-33C9* dsRNA solutions did not influence RNAi. Our results showed that silencing of the *cyp-33C9* gene decreased feeding and reproduction of *B. xylophilus*. In order to shed further light on the influences of *cyp-33C9* gene on *B. xylophilus*, we tested the effects of RNAi on the oviposition, percentage hatch and individual body length of *B. xylophilus* after silencing the *cyp-33C9* gene, to determine whether knockdown of the *cyp-33C9* gene affected growth and development of *B. xylophilus*. 

Eggshell formation is one of the most important factors affecting the hatching characteristics of phytoparasitic nematodes. Previous studies had shown that the eggshell of phytoparasitic nematodes is a structure with three crucial layers: vitelline layer, chitin layer and lipid-rich layer [35,36,37]. Benenati et al. studied the functions of *cyp-31A2* and *cyp-31A3* genes in *C. elegans,* using RNA-interference technology, and they discovered that both of these genes played an essential role in the development of the eggshell, especially the lipid-rich layer [17]. Their studies also showed that simultaneous depletion of *cyp-31A2* and *cyp-31A3* genes of *C. elegans* resulted in osmotic imbalance within the egg, impaired establishment of polarity and incorrect execution of meiosis, which eventually led to the death of *C. elegans* embryos. Interestingly, their findings mirrored those of Piano et al. who reported that the silencing of the *cyp-31A2* gene of *C. elegans* caused embryonic lethality [38]. 

Cytochrome P450 genes also play an important role in the oviposition and egg hatch of the parasite. In this study, our results showed that the number of eggs laid per female *B. xylophilus* was significantly lower after the *cyp-33C9* gene was silenced, and that approximately 50% of the *B. xylophilus* eggs produced were not capable of hatching. These results indicated that *cyp-33C9* gene had a marked effect on reproduction by negatively impacting both the egg-laying capacity and the percentage egg hatch of *B. xylophilus*. The explanation for this may be that, at the early stage of *B. xylophilus* embryo development, the *cyp-33C9* gene was required for formation of the lipid-rich layer of the embryo. The absence of the lipid-rich layer would have resulted in an inappropriate construction of the eggshell, which would then affect the egg-laying capacity of *B. xylophilus* [17]. Meanwhile, deletion of the *cyp-33C9* gene of *B. xylophilus* appeared to result in abnormal cell division and cortical polarization of eggs, which consequently resulted in a decline in the number of hatched eggs of *B. xylophilus* [17]. In the present study, knockdown of the *cyp-33C9* gene of *B. xylophilus* may be responsible directly for the abnormal development of the eggshell, which represents the permeability barrier for the embryos, ultimately leading to the decrease in oviposition number and percentage hatch of *B. xylophilus* eggs. In addition, cytochrome P450s have been reported to play important roles in hormone biosynthesis and metabolism [39]. The inhibited growth (as body length) and reproduction exhibited by the *cyp-33C9*-silenced *B. xylophilus* may be because the silencing of the *cyp-33C9* gene inhibited the biosynthesis of hormones. The data from the present study showed that silencing of the *cyp-33C9* gene, reduced the individual body length of *B. xylophilus* by 5–10%, albeit not significantly. It has been reported that the body size of *B. xylophilus* is driven by somatic polyploidy and cell proliferation simultaneously [40]. Consequently, we speculate that the *cyp-33C9* gene of *B. xylophilus* appears to have little effect on the polyploidization and proliferation of somatic cells. 

After nematodes invade a plant, the host will generate a wide range of nematicidal and nematistatic substances as part of a natural resistance response to attempted infection by the nematodes [41,42]. As a consequence, the nematodes must resist or metabolize these secondary metabolites in order to invade the host successfully [23,43]. Cytochrome P450s play an important role in the metabolism of xenobiotic compounds in general, and this activity may enable *B. xylophilus* to overcome the host resistance. Our data showed that, after the knockdown of the *cyp-33C9* gene of *B. xylophilus*, the percentage needle wilting of the *P. thunbergii* seedlings was markedly lower than that induced by the control nematodes. This result indicated that the *cyp-33C9* gene may play an important role in the pathogenic process of *B. xylophilus*. A possible reason for this may be that silencing of the *cyp-33C9* gene impaired the metabolism of xenobiotic compounds, subsequently slowing the migration of the nematodes and delaying the time course of wilting by the infected trees [44,45]. 

When *B. xylophilus* J2s had been soaked in *cyp-33C9* dsRNA, the nematodes took a longer time to migrate through the host trees (Table 1). Silencing of the *cyp-33C9* gene with RNAi decreased the number of nematodes in *P. thunbergii* seedlings compared with the control seedlings (Table 1). The results obtained by this study demonstrated that the knockdown of the *cyp-33C9* gene interrupted the invasiveness, fitness, motility or reproduction of *B. xylophilus* during invasion of the host. These results are consistent, in part, with those reported by Cheng et al. who discovered that silencing of the *Bx-eng-1* gene reduced the migration ability of *B. xylophilus* in *Pinus bungeana* [32]. 

In summary, this study investigated the function of the *cyp-33C9* gene of *B. xylophilus* by dsRNA interference. The results showed that the *cyp-33C9* gene could be effectively silenced by the dsRNA soaking method. We found that the silencing of the *cyp-33C9* gene suppressed feeding, reproduction, oviposition, percentage hatch and pathogenicity of *B. xylophilus*. However, silencing of the *cyp-33C9* gene had no significant effect on the individual body length of *B. xylophilus*. The migration speed of *B. xylophilus* treated with cyp-33C9 dsRNA was reduced in the early stages when nematodes were inoculated into pine trees. These data provide fundamental information to increase our understanding of pine wilt disease (PWD) and may help to develop effective strategies to prevent and control PWD. However, further experiments are needed to investigate the molecular mechanisms by which the *cyp-33C9* gene influences eggshell formation, and the correct execution of meiosis and polarization of the embryo.

## 4. Materials and Methods

### 4.1. Biological Materials

The BxJJ01 strain of *B. xylophilus* was isolated using Baermann funnels from infested *P. thunbergii* samples collected in Jiujiang City, Jiangxi Province, China. The nematodes were cultured on PDA plates covered with *B. cinerea* for five days at 25 °C. Subsequently, the nematodes were isolated from the plates with Baermann funnels and centrifuged at 1500× *g* for 5 min. About 10,000 mixed-stage nematodes were decanted into a burette, which contained 25 mL 0.3% carboxymethyl cellulose, sodium salt (CMC) solution. After 12 h, the second-stage larvae were collected from the top of the burette [46].

Two-year-old *P. thunbergii* seedlings used in this study were obtained from a forest farm in Jiujiang City and were transplanted into pots (18 cm in diameter, 11 cm in height), which were watered on alternate days. The heights of the seedlings were 40–50 cm and they were grown in an air-conditioned greenhouse with a relative humidity of 70% at 30 °C during the daytime and 25 °C at night.

### 4.2. RNA Isolation and cDNA Synthesis of Bursaphelenchus xylophilus

The total RNA was extracted from nematodes using RNeasy Mini Kit (Qiagen, Valencia, CA, USA) and purified with the RNAclean Kit (Tiangen, Beijing, China), following the manufacturer’s instructions. The total RNA concentration was detected at 260 nm using an ultraviolet spectrophotometer and its quality examined by electrophoresis on a 1% agarose gel. RNA was reverse transcribed to cDNA using a PrimeScript 1st strand cDNA Synthesis Kit (TaKaRa, Shuzo, Japan), according to the manufacturer’s instructions.

### 4.3. Double-stranded RNA (dsRNA) Synthesis and in Vitro RNA Interference (RNAi)

A fragment of *cyp-33C9* gene (1200-bp) was amplified by PCR from the cDNA templates of pine wood nematodes with the following primers: forward primer 5′-ACTTTCCTGGTAACACTG-3′ and reverse primer 5′-CTTTGATTCTTTGGACGA-3′. The *cyp-33C9* fragments were cloned into Pmd^TM^18-T vector and then amplified by PCR using T_7_-labeled gene-specific primers as follows: forward primer 5′-TAATACGACTCACTATAGGGACTTTCCTGGTAACACTG-3′ and reverse primer 5′-TAATACGACTCACTATAGGGCTTTGATTCTTTGGACGA-3′. The amplified products were used as template for synthesis of the single-strand RNA (ssRNA). The ssRNA was synthesized using a 20 μL reaction volume, which contained 2 μL template, 2 μL 10× T7 reaction buffer, 2μL ATP solution, 2 μL CTP solution, 2 μL GTP solution, 2 μL UTP solution, 2 μL T7 enzyme mix, with nuclease-free water added to a final volume of 20 μL. The ssRNAs were mixed together and incubated at 75 °C for 5 min to synthesize dsRNA. In addition, the dsRNA of green fluorescent protein gene (*gfp*) was synthesized with the following primers: forward primer 5′-GTACTCGAGTGGGTTATGGTGTTCTATGCT-3′ and reverse primer 5′-GAATCTAGAGTGGTCTCTCTTTTCGTTGG-3′ [32]. Then, the dsRNA was purified to remove proteins, free nucleotides and nucleic acid degradation products. Approximately 5000 J2 nematodes were immersed in 500 μL *cyp-33C9* dsRNA solution (1 μg·μL^−1^) and incubated in a shaking incubator at 180 rpm for 24 h at 25 °C. Equal numbers of nematodes immersed in *gfp* dsRNA solution or ddH_2_O without dsRNA were treated as controls. There were three replicates for each treatment. Samples from each treatment were washed with sterile water three times before use and the nematodes were collected by centrifuging at 1500× *g* for 3 min.

### 4.4. Quantitative Reverse Transcription PCR (qRT-PCR)

Quantitative reverse transcription PCR (qRT-PCR) was used to assess the effect of RNAi on *cyp-33C9* mRNA levels. Total RNA was extracted from *B. xylophilus* following *cyp-33C9* dsRNA treatment or ddH_2_O or *gfp* dsRNA controls. The first-strand cDNA was synthesized and used as a template for PCR. qRT-PCR was carried out using TransStart Green qPCR SuperMix (Trans GEN Biotech, Beijing, China) in a thermal cycler (ABI Prism 7500; Applied Biosystems, Foster City, CA, USA). The primers for the *cyp-33C9* gene were as follows: forward primer 5′-TCGGTTGTGGCGTGGATG-3′ and reverse primer 5′-TGAATTATGTTCAGGCGGTT-3′. The thermocycler conditions comprised one cycle of denaturation at 94 °C for 30 s, followed by 35 cycles at 94 °C for 5 s, 60 °C for 34 s, and 72 °C for 1 min. The actin gene of *B. xylophilus* was used as the internal control. The primers for the actin gene were as follows: forward primer 5′-GCAACACGGAGTTCGTTGTA-3′ and reverse primer 5′-GTATCGTCACCAACTGGGAT-3′. The experiment had three biological replicates (with respect to independent RNA preparations) and three technical replicates. 

### 4.5. Effect of RNA Interference (RNAi) on Feeding and Reproduction of Bursaphelenchus xylophilus

Two hundred nematodes (second-stage juveniles, J2s) were soaked in ddH_2_O (CK), *gfp* dsRNA solution or *cyp-33C9* dsRNA solution for 48 h, respectively. The nematodes were washed three times with sterile water. Subsequently, the nematodes were transferred onto a culture of *B. cinerea* on a PDA plate and cultured at 25 °C for 5 d. There were three replicates for each treatment. Feeding of *B. xylophilus* on *B. cinerea* was observed and photographed. Then, the nematodes were isolated from the PDA plates using Baermann funnels and counted with an optical microscope (CX31; Olympus, Tokyo, Japan) [47].

### 4.6. Effect of RNA Interference (RNAi) on Oviposition of Bursaphelenchus xylophilus

Approximately one thousand J2 nematodes were soaked in *cyp-33C9* dsRNA solution, ddH_2_O or *gfp* dsRNA solution for 48 h. After soaking, the nematodes were washed three times with sterile water and transferred to *B. cinerea* cultures on PDA plates in the dark at 25 °C for 24 h. Ten pairs of each of female and male adult nematodes were selected and mixed together for mating in small petri dishes (30-mm diameter) in the dark at 25 °C for 12 h. Subsequently, the resulting eggs laid by female nematodes were counted under an optical microscope (CX31; Olympus, Tokyo, Japan). Each treatment was replicated three times. The effects of the three different treatments on the egg-laying capacity of *B. xylophilus* were determined by comparing the number of eggs laid per female nematode. 

### 4.7. Effect of RNA Interference (RNAi) on Percentage Hatch of Bursaphelenchus xylophilus

In order to obtain synchronous eggs, adult nematodes were transferred to petri dishes (3 cm) where the depth of sterile water was less than 5 mm. After 0.5 to 1.0 h, some nematodes had laid eggs and most of the eggs were stuck to the bottom of the petri dish. By gently removing the water and nematodes to another petri dish, the synchronous eggs were collected by washing the petri dish several times with sterile water. Subsequently, one hundred eggs were soaked in *cyp-33C9* dsRNA solution, ddH_2_O or *gfp* dsRNA solution at 25 °C for 48 h. Each treatment was replicated three times. If an egg hatched within 36 h, the egg was considered to have hatched successfully; otherwise, an unhatched embryo was considered to be dead [48]. 

### 4.8. Effects of RNA Interference (RNAi) on Individual Body Length of Bursaphelenchus xylophilus

Two hundred J2 nematodes were soaked in *cyp-33C9* dsRNA solution, ddH_2_O or *gfp* dsRNA solution for 48 h. After soaking, the nematodes were washed three times with sterile water and transferred to *B. cinereal* cultures on PDA plates in the dark at 25 °C for 24 h. The nematodes were isolated from the plates with Baermann funnels and centrifuged at 1500× *g* for 5 min. Subsequently, nematodes were killed by warming up the solution to 55 °C. The nematodes were washed three times with sterile water and the individual body length of adult nematodes was measured under an optical microscope (CX31; Olympus, Tokyo, Japan). Forty female and forty male nematodes were measured for each treatment.

### 4.9. Effects of RNA Interference (RNAi) on Migration and Reproduction of Bursaphelenchus xylophilus in Pinus thunbergii

Approximately two thousand J2 nematodes soaked in *cyp-33C9* dsRNA solution, *gfp* dsRNA solution or ddH_2_O (as control) for 48 h were inoculated into *P. thunbergii* seedlings. For each treatment, three *P. thunbergii* seedlings were cut for observation every 5 d. After removing the branches and leaves, the trunks of *P. thunbergii* seedlings were cut into 5-cm long sections in either direction from the site of inoculation. Subsequently, *B. xylophilus* in the trunks of *P. thunbergii* were collected by Baerman funnels and counted under an optical microscope (CX31; Olympus, Tokyo, Japan) [34]. 

### 4.10. Effects of RNA Interference (RNAi) on Pathogenicity of Bursaphelenchus xylophilus

The 2-year-old *P. thunbergii* seedlings were inoculated with three different preparations at about 15–20 cm above the soil level, and the preparations were as follows: (i) 200 µL suspension of nematodes soaked in ddH_2_O; (ii) 200 µL suspension of nematodes treated with *cyp-33C9* dsRNA; (iii) 200 µL suspension of ddH_2_O without nematodes. The pine trees inoculated with *B. xylophilus* soaked in ddH_2_O or inoculated with ddH_2_O without nematodes were utilized as controls. Wilting symptoms of *P. thunbergii* were observed after inoculation for 25 d. A *P. thunbergii* seedling was deemed to be dead if all its needles turned yellow. Each treatment group contained nine *P. thunbergii* seedlings. The percentage wilting was calculated using the following equation [49]:

(1) $$\mathrm{The}\;\mathrm{percentage}\;\mathrm{wilting}\;=\frac{\sum \mathrm{Number}\;\mathrm{of}\;\mathrm{wilted}\;\mathrm{trees}}{\mathrm{Total}\;\mathrm{number}\;\mathrm{of}\;\mathrm{trees}}\;\times 100\%$$

### 4.11. Statistical Analysis

The results shown are the means and standard deviation (SD) of three replicates. Statistical analysis was determined using SPSS Statistics 13.0 software (IBM, Armonk, NY, USA), using one-way analysis of variance, with multiple pairwise comparisons conducted by Tukey’s test.

## 5. Conclusions

In summary, this study investigated the function of the *cyp-33C9* gene of *B. xylophilus* by dsRNA interference. The results showed that the *cyp-33C9* gene could be effectively silenced by the dsRNA soaking method. We found that the silencing of the *cyp-33C9* gene suppressed feeding, reproduction, oviposition, percentage hatch and pathogenicity of *B. xylophilus*. However, silencing of the *cyp-33C9* gene had no significant effect on the individual body length of *B. xylophilus*. The migration speed of *B. xylophilus* treated with cyp-33C9 dsRNA was reduced in the early stage when nematodes were inoculated in pine trees. These data provide fundamental information to increase our understanding of pine wilt disease (PWD) and may help to develop effective strategies to prevent and control PWD. However, further experiments are needed to investigate the molecular mechanisms by which the *cyp-33C9* gene influences eggshell formation, and the correct execution of meiosis and polarization of the embryo.

## Figures and Tables

**Table 1:** Effects of RNA interference on migration and reproduction of *Bursaphelenchus xylophilus* after inoculation into *Pinus thunbergii.*

Distance	Treatment	5 d	10 d	15 d	20 d	25 d
Up-5 cm	CK	66 ± 8a	458 ± 23a	4557 ± 314a	15459 ± 531a	16490 ± 325a
	*cyp-33C9*	29 ± 3b	122 ± 15b	967 ± 66b	7222 ± 253b	15423 ± 535a
	*gfp*	65 ± 5a	425 ± 28a	4224 ± 406a	14544 ± 854a	16117 ± 805a
Up-10 cm	CK	0	150 ± 12a	2350 ± 278a	16102 ± 438a	13445 ± 154a
	*cyp-33C9*	0	39 ± 5b	371 ± 19c	7903 ± 346b	13591 ± 260a
	*gfp*	0	138 ± 21a	1789 ± 209b	14711 ± 1072a	13731 ± 439a
Up-15 cm	CK	0	78 ± 7a	1482 ± 36a	6533 ± 322a	1328 ± 62a
	*cyp-33C9*	0	13 ± 1b	187 ± 9b	2326 ± 172b	1299 ± 69a
	*gfp*	0	71 ± 7a	1487 ± 116a	6881 ± 224a	1343 ± 73a
Down-5 cm	CK	256 ± 30a	867 ± 56a	6799 ± 312a	14948 ± 416a	13086 ± 449a
	*cyp-33C9*	84 ± 11b	392 ± 32b	5634 ± 109b	15071 ± 261a	12764 ± 422a
	*gfp*	246 ± 43a	889 ± 30a	6365 ± 385ab	15503 ± 428a	13272 ± 413a
Down-10 cm	CK	57 ± 6a	435 ± 20a	7510 ± 303a	16239 ± 338ab	13707 ± 129a
	*cyp-33C9*	22 ± 3b	194 ± 11b	6512 ± 223b	15669 ± 210b	12871 ± 390b
	*gfp*	59 ± 3a	447 ± 35a	7834 ± 307a	16579 ± 305a	13255 ± 310ab
Down-15 cm	CK	9 ± 1a	152 ± 13a	3222 ± 169a	13247 ± 382a	10447 ± 724a
	*cyp-33C9*	0	42 ± 3b	1282 ± 47b	8246 ± 208b	7233 ± 533b
	*gfp*	7 ± 2a	149 ± 7a	3145 ± 194a	12877 ± 380a	9099 ± 514a

The numbers in the first column show the distance from the inoculation sites. The other numbers in the table represent the number of *B. xylophilus* from the different treatments which were recovered at each site after inoculation of 2000 s-stage juveniles at each inoculation site. The data represent mean values ± standard deviation (SD) from three independent experiments. Different lowercase letters indicate significant differences (*p* < 0.05), using Tukey’s test, among treatments.
